# Ophthalmology Consultation Plan in the Context of 2019-nCoV

**DOI:** 10.3389/fmed.2021.618599

**Published:** 2021-05-12

**Authors:** Shuang Song, Yidan Chen, Ying Han, Feng Wang, Ying Su

**Affiliations:** ^1^Department of Ophthalmology, The First Affiliated Hospital of Harbin Medical University, Harbin, China; ^2^Department of Geriatric, The First Affiliated Hospital of Harbin Medical University, Harbin, China

**Keywords:** SARS-CoV-2, ophthalmology, consultation, transmission, guidelines

## Introduction

Since December 2019, new coronavirus pneumonia (2019-nCoV) has been spread globally. At first it concentrated outbreak in China, and now reported all over the world, particularly in America and Europe (1, 2). 2019-nCoV is a new virus that everyone can be infected and was labeled as a pandemic by the World Health Organization (WHO) (3). A study in Wuhan reported of 535 patients, 27 patients (5.0%) presented with conjunctival congestion and 4 patients had conjunctival congestion as the initial symptom (4). Besides, a recent paper found that Several infected cases presented firstly with conjunctivitis before the onset of pneumonia, implying that the ocular route might be the potential transmission route of SARS-CoV-2 virus under certain conditions (5). An effective barrier protecting the eye is of paramount importance because the droplets and body fluids have a high probability to get onto the conjunctiva surface (6). Hence safety goggles identified to reduce ocular transmission but ophthalmologists need to close contact with the patients (conjunctival, tear secretions and aerosol secretions) during the fundus examination and other operations, so Ophthalmologists are a high-risk category (7, 8). For large medical centers their daily outpatient clinic and emergency lists have a high patient volume, which also increases the risk of cross-transmission between medical staff and patients (7, 8).

Due to different national conditions and cultures, the measures to respond to the pandemic are also different, so summary the diagnosis and treatment experience of 2019-nCoV and formulating a guideline suitable for the diagnosis and treatment of ophthalmic patients have very important clinical significance for epidemic prevention and control. In this review, we have combined our two experiences in fighting 2019-nCoV and the latest literature to propose rational recommendations for patients with eye diseases during the epidemic, and provide reference opinions for ophthalmology clinics and surgical procedures.

## Advice for Ophthalmic Patients

As a regional eye diagnosis and treatment center, our outpatient waiting room is often overcrowded. Risk assessment of patients to achieve patient diversion and avoiding the gathering of patients in large hospitals is an effective means to reduce human-to-human transmission. At this time, the patient needs to know what kind of eye diseases can be treated conservatively at home? Doctors also need to know which eye diseases can be recommended for patients to treat at home when the patient consults? During the epidemic period according to our experience, ophthalmic diseases that can be treated later include (8–11):

- Refractive errors, strabismus and amblyopia. For example, the child's refractive error is usually checked every 6 months. During the epidemic, the check can be post-poned, but if the check is necessary; parents can stagger the holidays because it is the peak period for consultation.
- Patients with eye discomfort such as dry eyes, foreign body sensation, but no eye pain, and vision loss can use artificial tears first.
- Ocular surfacepartial bleeding without other symptoms may be subconjunctival hemorrhage, just observe.
- The long-term gradual decline of visual without pain in the elderly may be age-related cataract, which does not change much in the short term;
- Poor vision, deformed vision, and no significant changes in recent symptoms (such as age-related macular degeneration);
- Glaucoma with good drug control;
- Floaters without obvious changes and without affecting vision.

During home isolation, if eye discomfort such as redness and itching occurs, you can consult an ophthalmologist free of charge on the official website, take medication under the guidance of a physician, and stay away from crowded hospitals based on the principle of maximum benefit. However, it is recommended to go to the doctor in time in the following situations (9, 12, 13):

- Moderate to severe eye trauma: such as chemical burns, thermal burns, eyeball ruptures, etc.
- Redness and pain with obvious loss of vision: This condition may be inflammation of the eye such as keratitis, glaucoma, and iritis.
- Painless sudden drop in vision: such as sudden loss of vision for no apparent reason, fixed black shadow in front of the eyes and gradually enlarged.

## Outpatient Management Plan

### Management of Outpatient Hygiene Environment

- Places that can be ventilated, open windows, 3 times a day, no <30 min each time, In a place without ventilation, irradiate with ultraviolet light twice a day, 30~60 min each time (14).
- The floor and wall should be cleaned with chlorine-containing disinfectant first. Use 1,000 mg/L to disinfect 3 times a day, each action is 30 min, and it will be disinfected at any time if it is contaminated; the contaminated area should be disinfected 3 times a day, and the action is 30 min (14–16).
- Seventy-five percentage ethanol wipe disinfection is the first choice for high-frequency contact surfaces, followed by wiping with 1,000 mg/L chlorine disinfectant, which is disinfected 3 times a day. The mobile phones of medical staff should be wiped frequently with 75% ethanol disinfectant, at least once a day (14–16).

### Medical Equipment Disinfection

- Use disposable medical equipment, instruments and articles as much as possible, and treat them as infectious medical waste after use (14).
- Reusable medical equipment: Depending on whether the reused medical equipment is corrosion-resistant or not, wipe with 75% ethanol disinfectant or soak with 1,000 mg/L chlorine disinfectant (14).

### Patient Management

**Figure 1:**
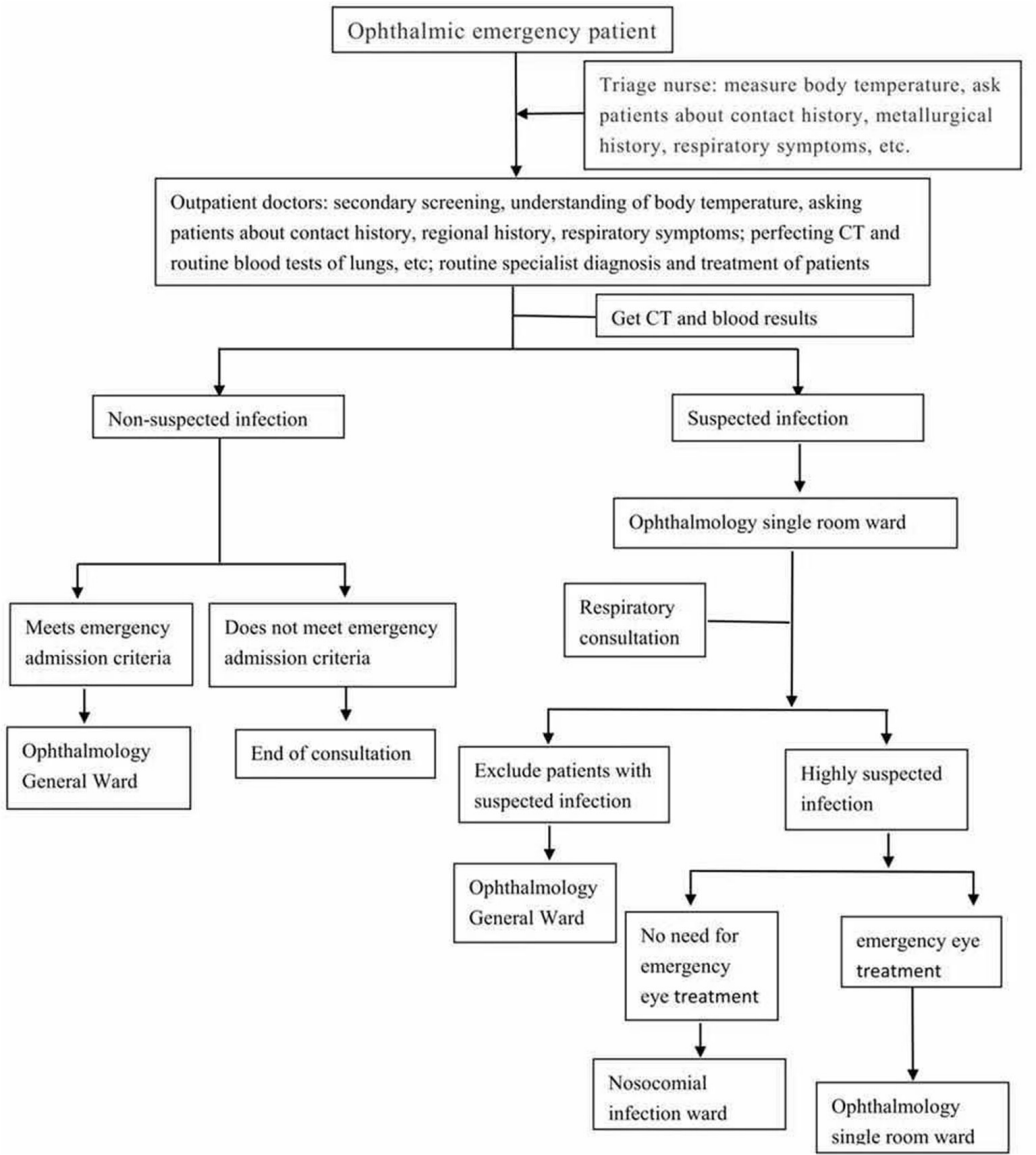
Diagnosis and treatment process of ophthalmic emergency patients.

- Ophthalmology patients register online to reduce queuing, and show a health QR code to prove that they have not been to high-risk areas.
- Everyone must wear a mask, measure the temperature, and disinfect their hands (8).
- Infrared fever detectors and thermometers (guns) are used to monitor the temperature of patients, and patients with fever are transferred to fever clinics (8, 11).
- Keep patients in the waiting area at least 1 m away from each other (11).
- Careful screening of the visiting patients as shown in Figure 1.

Patients who require oral or topical drugs after consultation can contact the doctor for consultation by phone or WeChat, tell your doctor about your condition, and guide medication through the network.

### Outpatient Staff Management

- Epidemic theory knowledge training, protective equipment use training (8).
- Strictly require hand hygiene.
- Correctly select the protection level according to the exposure risk classification.
- Handling abnormalities of protective equipment quickly (such as damaged protective clothing, damaged gloves, fogging of goggles, loose masks) (14).

### Special Attention of Ophthalmologists

Since the ophthalmologists in the outpatient clinic will inevitably come into contact with the patient's body fluids during the diagnosis and treatment (such as fundus examination), we must pay more attention to self-protection than ordinary staff.

- Ophthalmologists need third-level protection [Work clothes, medical protective clothing (disposable), isolation gowns, medical protective masks, work caps, protective visor/goggles, double gloves, hand hygiene] (8, 17, 18).
- Wash hands before and after treating patients.
- One room, one doctor and one patient. Strictly limit the number of people in the outpatient clinic (8).
- It is best for ophthalmologists to wear a face barriers instead of goggles, because the face barriers can well isolate the patient's body fluids or respiratory droplets during close examinations.
- It is best to use a non-contact tonometer or single-use disposable tonometer tip, and it is better to use indirect inspection methods rather than direct contact with the patient. All instruments used must be cleaned with 75% alcohol or chlorine-containing disinfectant in time (17, 18).
- A protective barriers can be added to inspection machines such as slit lamps and clean the contamination on the patient side in time (17, 18).
- Keep the examination limited to that required to make a clinical decision special tests requested (visual field, optical coherence tomography, corneal topography, ultrasound) only when critical to making a clinical decision.

Doctor Wang, a member of the national expert panel on pneumonia, was infected with 2019-nCoV in Wuhan (5). He wore a N95 mask but did not wear anything to protect his eyes. A few days before the onset of pneumonia, he developed redness and other conjunctival infections. Therefore, it is highly suspected that 2019-nCoV is first invaded by the conjunctiva, leading to respiratory infections. In addition, ophthalmologists have many inspections and operations during the diagnosis and treatment, and the distance between the doctor and the patient is very close during the inspection (8). The above reasons lead to a higher risk of infection by the ophthalmologist, and any improper protection is extremely easy to infect. Therefore, strict screening of patients is necessary at the time of their visit, and at the same time try to reduce the relevant examinations as much as possible, and doctors must perform secondary protection for necessary inspections and tertiary protection for high-risk operations. In the face of new coronary pneumonia, any prudent self-protection by ophthalmologists is reasonable.

### Surgical Procedure for n-2019-nCoV Patients

In principle, only emergency ophthalmic surgery is performed during the epidemic, limited-period surgery should delay the operation time. During the hospitalization period, minimize the number of escorts and reduce the flow of personnel. All hospitalized patients and escorts need nucleic acid test for pathogenic screening of new coronary pneumonia.

### Management of Ophthalmology Ward

- Set up general departments and isolation departments to isolate patients suspected or diagnosed with new coronavirus pneumonia (8, 14).
- Buffer wards are set up in normal departments for suspicious patients.
- If suspected or confirmed patients are found in the ward, relevant emergency plans and work procedures will be activated and transferred to a specific area for treatment (18).
- All hospitalized patients are checked for 2019-nCoV nucleic acid, questionnaire about upper respiratory symptoms, fever, myalgia and anosmia, domicile or traveling in hot areas, and contact history with confirmed or suspected COVID-19 patients within the past 14 days (8, 19).
- Hygienic environment of the normal ward reference 3.1

### Pre-operative Management

- Not infected patients are normally prepared for eye surgery.
- For patients who are diagnosed or suspected and must be operated, an independent negative pressure operating room should be arranged. If there is no negative pressure operating room, an independent clean room with relatively independent spatial location should be selected (8, 14, 19).
- If 2 or more suspected or confirmed infections occur at the same time, surgery should be performed for the more critically ill patients (19).
- Disposable medical supplies are preferred. Non-disposable equipment and items must have a clear cleaning and disinfection process.
- Perform detailed inspections of surgical supplies before surgery to reduce the activities of personnel during surgery.

### Intraoperative Management

- Most ophthalmic operations are not general anesthesia, patients can continue to wear surgical masks during the operation; if it is general anesthesia, it is recommended to place a disposable filter between the tracheal intubation and the breathing circuit to reduce the pollution of the breathing circuit, the anesthesia machine Strictly disinfect after use (20).
- Ordinary goggles seriously impede microsurgery, replaced it with homemade goggles by sealing the own glasses or flat lenses around the eyes with plastic wrap and remove the plastic wrap from the center of the lens to get protection and clear vision during the ophthalmic microsurgery (21, 22).
- Protective shields can be added to the slit lamp microscope to reduce the risk of close face-to-face contact between doctors and patients (21–23).
- Adjust the examination light from weak to strong, increasing gradually to avoid tears or a reflex sneeze.
- The third level of protection for surgeons in the whole process: hand-brushing clothing, medical protective clothing (disposable), surgical gown (disposable), medical protective masks, work caps, protective visor/goggles, double gloves (23).
- The number of people in the room is limited to the minimum number of patients required for care and surgery.

### Post-operative Management

- Medical staff escorting the patient should do a tertiary level of protection, and the patient must wear a mask all the way.
- A special transfer flat car should be used to achieve “one person, one use, one disinfection,” and the transfer elevator should also be disinfected on the surface. During the transfer of patients, attention should be paid to the protection of public areas and public appliances. Transfer patients according to the transfer route specified by the hospital and the special transfer route in the negative pressure operating room (14, 19).
- Disposal of surgical supplies, reusable medical supplies are placed separately and transported to disinfection supply center for centralized processing. Disposable items used in patients need to be handed over separately, delivered directly, and processed uniformly. A new coronavirus label is prominently placed on the outside of the pathological specimen bag and delivered by special personnel (14, 19).

### Isolation Observation of Medical Staff

All medical staff involved in the operation of suspected or infected patients should be isolated for medical observation after surgery. If the suspected case excludes infection, release the isolation of the surgical staff; if it is an infected person, continue to isolate and observe until 14 day. And pay attention to observe whether the clinical manifestations of the new coronary pneumonia mentioned above, and report to the competent department.

## Summary

2019-nCoV is a global biochemical crisis. Ophthalmologists should remind patients to pay attention to eye protection and hand hygiene to prevent the eyes from becoming a gateway to viral infection. Prevent mutual infection between patients and patients, and between patients and doctors, disinfection and isolation of departments and operating rooms, and establish a simple and effective diagnosis and treatment process is necessary. In this review, we clearly told patients with eye diseases which conditions can be post-poned for medical treatment, which conditions require urgent medical attention, and provide our experience on the protective measures in the outpatient and ward. We have experienced two local outbreaks of the epidemic, and the infection rate is zero. It is difficult to determine which method is the most important, but in the face of infectious diseases, every detail is very important. China's success in fighting the epidemic shows that these methods are effective. In the context of the normalization of the global epidemic, we hope that our experience can help ophthalmologists.

## Author Contributions

FW designed the article. YS drafted important content. SS wrote the manuscript. YC and YH provided important intellectual conment during revising the article. All authors listed have made a substantial, direct and intellectual contribution to the work, and approved it for publication.

## Conflict of Interest

The authors declare that the research was conducted in the absence of any commercial or financial relationships that could be construed as a potential conflict of interest.
